# Monitoring Spontaneous Quiescence and Asynchronous Proliferation-Quiescence Decisions in Prostate Cancer Cells

**DOI:** 10.3389/fcell.2021.728663

**Published:** 2021-12-10

**Authors:** Ajai J. Pulianmackal, Dan Sun, Kenji Yumoto, Zhengda Li, Yu-Chih Chen, Meha V. Patel, Yu Wang, Euisik Yoon, Alexander Pearson, Qiong Yang, Russell Taichman, Frank C. Cackowski, Laura A. Buttitta

**Affiliations:** ^1^ Molecular, Cellular and Developmental Biology, University of Michigan, Ann Arbor, MI, United States; ^2^ School of Dentistry, University of Michigan, Ann Arbor, MI, United States; ^3^ Department of Biophysics, University of Michigan, Ann Arbor, MI, United States; ^4^ Department of Electrical Engineering and Computer Science, University of Michigan, Ann Arbor, MI, United States; ^5^ Department of Computational and Systems Biology, Hillman Cancer Center, University of Pittsburgh School of Medicine, Pittsburgh, PA, United States; ^6^ Department of Biomedical Engineering, University of Michigan, Ann Arbor, MI, United States; ^7^ Center for Nanomedicine, Institute for Basic Science (IBS) and Graduate Program of Nano Biomedical Engineering (Nano BME), Advanced Science Institute, Yonsei University, Seoul, Korea, South Korea; ^8^ Department of Medicine, Section of Hematology/Oncology, University of Chicago Medical Center, Chicago, IL, United States; ^9^ Department of Periodontology, School of Dentistry, University of Alabama at Birmingham, Birmingham, AL, United States; ^10^ Department of Oncology, Karmanos Cancer Institute and Wayne State University, Detroit, MI, United States

**Keywords:** quiescence, dormancy, prostate cancer, cell cycle, live imaging

## Abstract

The proliferation-quiescence decision is a dynamic process that remains incompletely understood. Live-cell imaging with fluorescent cell cycle sensors now allows us to visualize the dynamics of cell cycle transitions and has revealed that proliferation-quiescence decisions can be highly heterogeneous, even among clonal cell lines in culture. Under normal culture conditions, cells often spontaneously enter non-cycling G0 states of varying duration and depth. This also occurs in cancer cells and G0 entry in tumors may underlie tumor dormancy and issues with cancer recurrence. Here we show that a cell cycle indicator previously shown to indicate G0 upon serum starvation, mVenus-p27K-, can also be used to monitor spontaneous quiescence in untransformed and cancer cell lines. We find that the duration of spontaneous quiescence in untransformed and cancer cells is heterogeneous and that a portion of this heterogeneity results from asynchronous proliferation-quiescence decisions in pairs of daughters after mitosis, where one daughter cell enters or remains in temporary quiescence while the other does not. We find that cancer dormancy signals influence both entry into quiescence and asynchronous proliferation-quiescence decisions after mitosis. Finally, we show that spontaneously quiescent prostate cancer cells exhibit altered expression of components of the Hippo pathway and are enriched for the stem cell markers CD133 and CD44. This suggests a hypothesis that dormancy signals could promote cancer recurrence by increasing the proportion of quiescent tumor cells poised for cell cycle re-entry with stem cell characteristics in cancer.

## Introduction

Cycling cells tend to enter quiescence, a reversible, non-cycling state in response to contact inhibition, reduced levels of mitogens, or under various stress conditions. Quiescent cells retain the ability to re-enter the cycle upon the addition of serum or under favorable conditions (Coller et al., 2006; Yao, 2014). However, studies of mammalian cells in the past few years have found that many cells enter a spontaneous reversible G0-like state in cell culture even in the presence of mitogens and abundant nutrients (Spencer et al., 2013; Overton et al., 2014; Min and Spencer, 2019). This suggests that the proliferation-quiescence decision is constantly regulated—even under optimal growth conditions.

The relative percentage of cells that enter a temporary G0-like state after mitosis varies with cell type and culture conditions, suggesting many signaling inputs influence the proliferation-quiescence decision (Spencer et al., 2013). This is also consistent with findings in several cancer cell lines, where some cells enter a temporary quiescent state while others do not (Dey-Guha et al., 2011). This leads to heterogeneity in cell culture, with a subpopulation of cells entering and leaving temporary quiescent states (Overton et al., 2014). This proliferative heterogeneity may underlie states of dormancy in cancer and has been shown to be related to cancer therapeutic resistance (Recasens and Munoz, 2019; Risson et al., 2020; Nik Nabil et al., 2021). This is particularly relevant in prostate cancer, where it is thought that early spreading of tumor cells to the bone marrow and other tissues may provide signals leading to quiescence and tumor dormancy (Chen et al., 2021). Prostate cancer dormancy in tissues such as the bone are problematic as a percentage of patients will later develop recurrent cancer with significant metastases from these cells, which are often also resistant to treatment (Lam et al., 2014). Understanding how and why quiescent cancer cells reside in environments such as the bone marrow for long periods of time, and finding ways to eliminate them, is an important ongoing challenge in prostate cancer research and treatment.

The difficulties in monitoring the proliferation-quiescence decision and distinguishing different states and lengths of G0 has limited our ability to understand how signals impact the heterogeneity of quiescence in cell populations. Most assays for cell cycling status use immunostaining of cell cycle phase markers or nucleotide analogue incorporation, both of which assess static conditions in fixed samples (Matson and Cook, 2017). Cell cycle reporters such as the FUCCI system (Fluorescent Ubiquitination-based Cell Cycle Indicator), have become widely used to track cell cycle dynamics live in individual cells (Sakaue-Sawano et al., 2008). The FUCCI system and related systems such as CycleTrak and others including a constitutive nuclear marker are able to differentially label cells in G1, S and G_2_/M phases, allowing us to visualize the G_1_-M transition, however G0 cannot be distinguished from G1 in these approaches (Ridenour et al., 2012; Chittajallu et al., 2015).

Recent methods to monitor quiescence heterogeneity have used live cell imaging with sensors for Cdk2 activity, Ki-67 expression, and expression of Cdk inhibitors such as p21 and p27 (Spencer et al., 2013; Overton et al., 2014; Stewart-Ornstein and Lahav, 2016; Miller et al., 2018; Zambon et al., 2020). Here we take advantage of the cell cycle indicator, mVenus-p27K^−^, which was generated to work in combination with the G0/G1 FUCCI reporter mCherry-hCdt1 (30/120), to specifically label quiescent cells (Oki et al., 2014). This probe is a fusion protein consisting of a fluorescent protein mVenus and a Cdk binding defective mutant of p27 (p27K-). p27 accumulates during quiescence and is degraded by two ubiquitin ligases: the Kip1 ubiquitination-promoting complex (KPC) at the G0-G1 transition, and the SCF^Skp2^ complex at S/G2/M phases (Kamura et al., 2004). When used in combination with the G0/G1 FUCCI reporter, cells can be tracked from a few hours after mitosis until early S phase with distinct colors. This allows us to examine the dynamics of the proliferation-quiescence transition after mitosis on a single-cell level without artificial synchronization.

Prior work with a Cdk2 sensor and monitoring p21 levels revealed that both non-transformed and cancer cells in culture can enter “spontaneously” quiescent states of variable length, even under optimal growth conditions (Spencer et al., 2013; Overton et al., 2014; Yang et al., 2017; Min and Spencer, 2019). The proportion of spontaneously quiescent cells in a population and their variability in the length of quiescence leads to cell cycle heterogeneity (Overton et al., 2014), which may in part also underlie cell cycle heterogeneity within clonal tumors (Dey-Guha et al., 2011; Dey-Guha et al., 2015). This led us to examine whether we could monitor spontaneous quiescence using the mVenus-p27K^−^ G0 reporter. Here we show that by tracking the trajectory of this reporter activity, we can monitor spontaneous quiescence in non-transformed mouse fibroblasts and prostate cancer cells. While measuring the heterogeneity of spontaneous quiescence, we also observed that a pair of daughter cells resulting from a single mitosis can make asynchronous proliferation-quiescence decisions. In this type of asynchronous decision, one daughter from a mitosis enters G0, while the other enters G1, further increasing cell cycle heterogeneity within a clonal population (Dey-Guha et al., 2011). We find that signals associated with promoting or releasing tumor dormancy can influence quiescence and asynchronous proliferation-quiescence decisions in prostate cancer cells. Using the mVenus-p27K^−^ G0 reporter, we isolate populations containing quiescent cancer cells and find they are enriched for a subpopulation expressing stem cell markers and express high levels of Hippo pathway signaling components, but with inactivated YAP, which may indicate a state poised for cell cycle re-entry. Finally, we provide evidence that the expression of immune recognition signals may be decreased in populations containing quiescent cancer cells, suggesting a hypothesis for how these cancer cells may preferentially evade the immune system.

## Results

### mVenus-p27K^−^ Based G0/G1 Cell Cycle Indicators Track Spontaneous Quiescence

**FIGURE 1:**
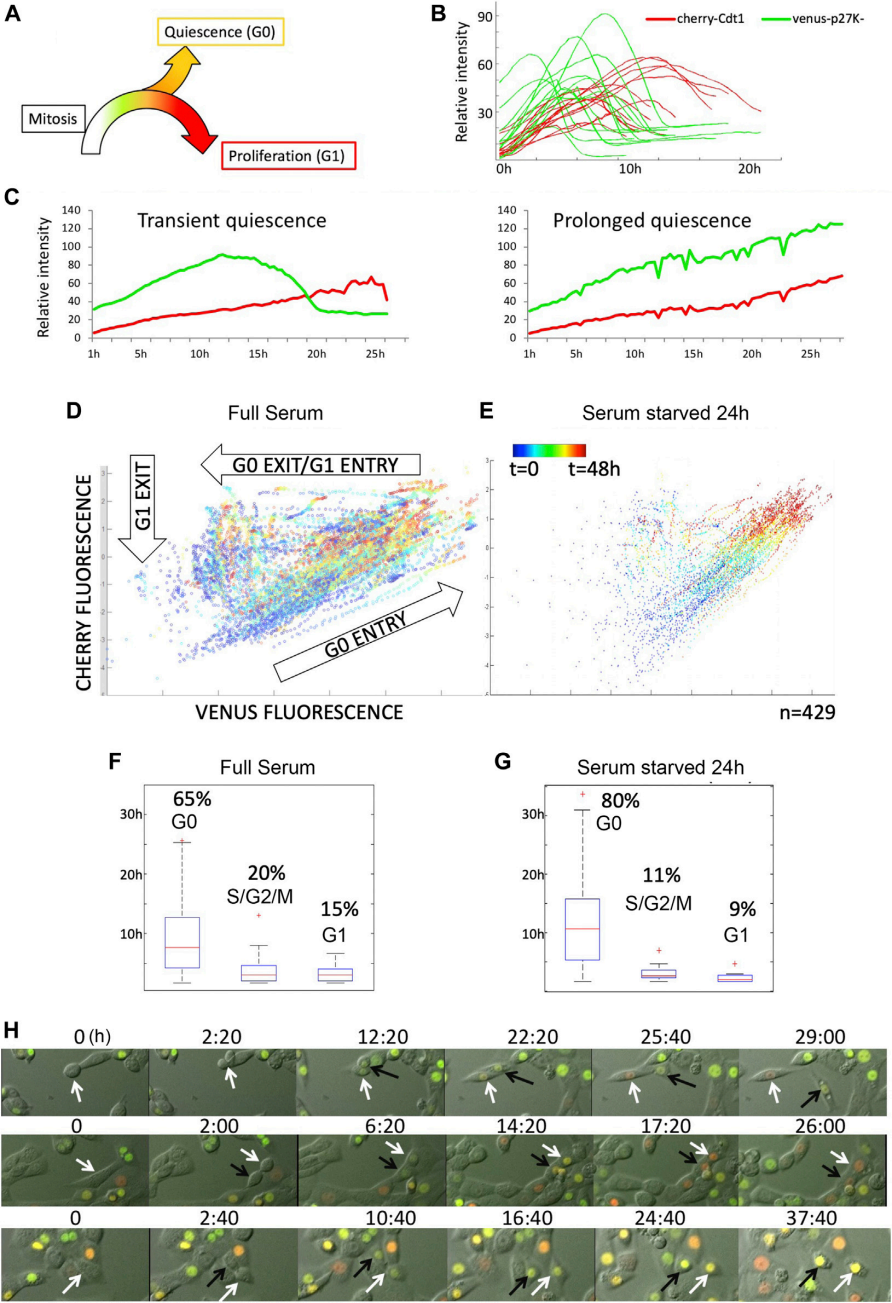
The G0 sensor, mVenus-p27K-, can be used to monitor spontaneous quiescence and asynchronous proliferation-quiescence decisions in untransformed cells. **(A)** The proliferation-quiescence decision as monitored with the G0 sensor, mVenus-p27K- (G0-Venus) and G1 sensor hCdt1_(30–120)_ -Cherry (G1-Cherry). For NIH/3T3 cells, on average, 2–4 h after cytokinesis, G0-Venus expression begins increasing, followed approximately 3–4 h later by G1-Cherry expression. For cells entering G1, the Venus/Cherry double-positive phase lasts 5–10 h. For quantification purposes we define a Venus/Cherry double-positive phase prolonged beyond 14 h as spontaneous G0. **(B)** Example traces of G0-Venus/G1-Cherry reporter dynamics in cells entering the cell cycle. 0 h is relative time, aligned to the start of G0-Venus reporter increase. **(C)** Example traces of G0-Venus/G1-Cherry reporter dynamics in cells under full serum conditions. Left shows a transient spontaneous G0 state of less than 15 h, while right shows an example of prolonged, spontaneous quiescence lasting over 24 h. **(D)** Cell trajectories followed over time from several movies show reporter behaviors consistent with G0 entry, G0 exit and G1 entry, and exit from G1 and early S-phase under full serum conditions. **(E)** Under serum starvation for 24 h, multiple trajectories collapse into G0 entry. **(F)** Under full serum conditions, time spent in G0 is highly variable. **(G)** Under serum starvation for 24 h G0 is prolonged. **(H)** Frames from movies showing examples of mitoses followed by an asynchronous G0/G1 decision **(top)**, synchronous G1 decision **(middle)** and synchronous G0 decision **(bottom)**.

To characterize the proliferation-quiescence transition at single-cell resolution in mouse 3T3 cells under full serum conditions without synchronization, we used the G0 cell cycle indicator mVenus-p27K^−^ combined with the G0/G1 reporter from the FUCCI cell cycle system, mCherry-hCdt1 (30/120) to distinguish G0 cells from G1 cells as previously described (Oki et al., 2014). We first manually examined movies of asynchronously proliferating 3T3 cells stably expressing these reporters to monitor reporter dynamics (Supplementary Movie S1, S2). With this combination of cell cycle reporters, mVenus-p27K^−^ expression begins approximately 2–6 h after cytokinesis is complete, followed by mCherry-hCdt1expression approximately 2–6 h later. Most cells then exhibit a rapid reduction in mVenus-p27K^−^ within approximately 3 h, signaling G1 entry followed by mCherry-hCdt1 degradation at G1 exit (Figures 1A,B). However, for a fraction of cells (ranging between 20–65% in different movies) we observed both mVenus-p27K^−^ and mCherry-hCdt1 to both continue to accumulate for up to 14 h and beyond, without division or evidence of S/G2/M entry for 20 h or more, signaling spontaneous G0 entry (Figure 1C). This progression of reporter expression in order from mVenus-p27K^−^ positive to mVenus-p27K^−^ and mCherry-hCdt1 double positive to mCherry-hCdt1 was invariant in the movies, although we did observe some cell to cell variation in reporter expression intensity, despite using a clonal cell line.

To monitor and quantitatively measure the dynamic transitions of cell cycle states—from cytokinesis to S phase entry, we developed an Automated temporal Tracking of Cellular Quiescence (ATCQ) analysis platform. This platform includes a computational framework for automated cell segmentation (identification of individual cells in an image), tracking, cell cycle state identification, and quantification from movies (Supplementary Figure S1). The cell segmentation and tracking allows us to record the fluorescent reporter intensity changes within individual cells in real-time imaging, without the aid of a constitutive nuclear marker. The single-cell fluorescence changes over time, in turn, are used to obtain cell cycle state identification (G0, G1, or early S phase) and quantification, which allows us to examine the kinetics of the proliferation-quiescence transition. The single-cell traces of fluorescent reporters, mVenus-p27K^−^ and mCherry-hCdt1, graphed by ATCQ is consistent with trajectories of G0 entry (increasing Venus and Cherry), G0 exit/G1 entry (degradation of Venus, increasing Cherry), and G1 exit/S-phase progression (degradation of Cherry) we manually observed in movies (Figure 1D).

To confirm that the Venus/Cherry-double positive population represents G0 phase, we performed a short-term (24 h, 1%FBS) serum starvation treatment followed by 48 h of live imaging. As expected in low serum, the reporter trajectories collapsed into predominantly G0 entry (Figure 1E). When we measure the timing of the reporter trajectories we find that the timing of G0 is heterogeneous compared to G1 entry and G1 exit and becomes further prolonged under low serum (Figures 1F,G).

We next examined whether the mVenus-p27K^−^/mCherry-hCdt1 double positive population under full serum conditions exhibits molecular markers of G0. To do this, we sorted cells into Venus/Cherry double-positive, Cherry single-positive, and Venus/Cherry double-negative populations and performed western blots for markers of G0 vs G1 phase. As a positive control for G0, cells cultured under serum deprivation were sorted in a similar manner (Supplementary Figure S2). We found that Venus/Cherry double-positive cells under full serum conditions exhibited hypo-phosphorylated pocket proteins RB and p130, increased endogenous p27, and reduced phosphorylation of Cdk2 on the activating T-Loop (Jeffrey et al., 1995; Sherr and Roberts, 1999; Tedesco et al., 2002). We also confirmed reduced expression of cell cycle genes, and upregulated expression of genes associated with G0 in the double positive cells in full serum by qRT-PCR on sorted cells (Supplementary Figure S2) (Oki et al., 2014). Taken together, our tracking and molecular data suggests that many of the Venus/Cherry double-positive cells under full serum conditions enter a temporary G0 of variable length. We therefore conclude that this reporter combination also captures temporary, spontaneous quiescence in a fraction of asynchronously proliferating cells.

### Asynchrony in the Proliferation-Quiescence Decision

In the manual tracking of dividing cells, we noticed several instances where pairs of daughters, born of a single mitosis, make different proliferation-quiescence decisions. In this situation, one daughter will remain G0, while the other daughter born at the same time will degrade the mVenus-p27K^−^ reporter and enter G1, followed by S/G2 and mitosis (Figure 1H). Under normal culture conditions we observe this in 20–40% of 3T3 cells entering G0, with the differences in the timing of G1 entry between asynchronous daughters varying from 1–15 h. We also find that daughters can exhibit varying lengths of the Venus/Cherry double-positive state (from 5—24 h) before one from the pair enters G1. We next examined whether such asynchrony in the cell cycle progression of two daughters born of the same mitosis could be observed in other cell types. We manually examined movies of published live cell imaging and observed instances of cell cycle asynchrony in pairs of daughters in BT549 and MCF10A cells (Supplementary Table S1). Cell cycle asynchrony in daughters born of the same mitosis has also been reported in MCF7 and HCT116 cells and referred to as “asymmetric” cell divisions, accompanied by differences in AKT signaling between daughters after telophase (Dey-Guha et al., 2011; Dey-Guha et al., 2015). We suggest that both spontaneous G0 and asynchronous proliferation-quiescence decisions in pairs of daughters after mitosis both contribute to cell cycle heterogeneity in clonal cell populations.

### PC3 Prostate Cancer Cells Exhibit Spontaneous Quiescence and Asynchrony in the Proliferation-Quiescence Decision

**FIGURE 2:**
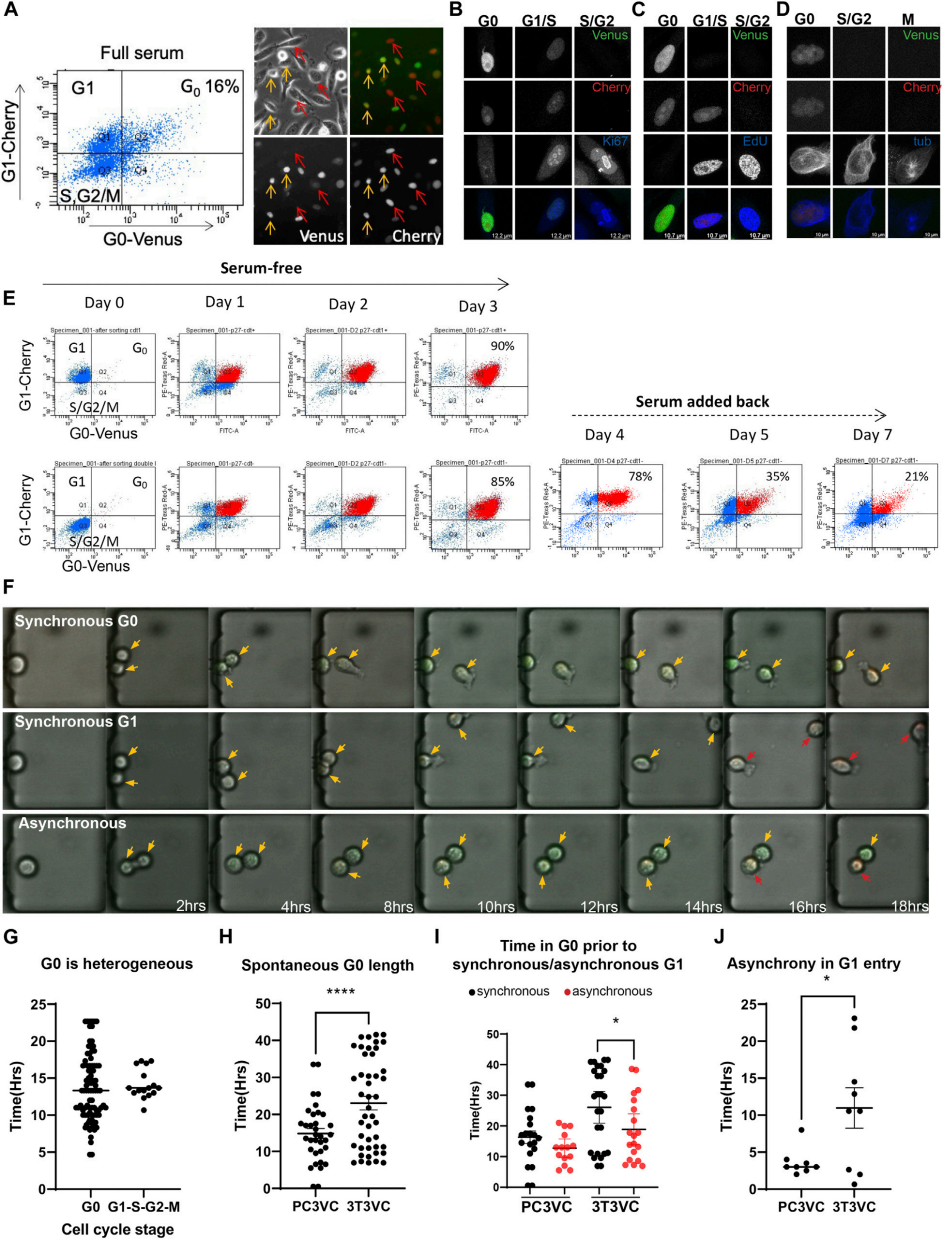
Spontaneous quiescence and asynchronous proliferation-quiescence decisions occur in PC3 cells. **(A)** G0-Venus and G1-Cherry reporters were transduced into PC3 cells and a clone exhibiting normal growth rate and strong, stable reporter expression was isolated. PC3 Venus/Cherry cells exhibit a fraction of cells double positive for G0-Venus/G1-Cherry under full serum conditions. Imaging reveals Venus/Cherry double positive cells (orange arrows), Cherry only positive cells (red arrows) and double negative cells (not indicated). **(B)** PC3 cells double positive for G0-Venus/G1-Cherry are Ki-67 negative, **(C)** EdU negative, **(D)** and cells in mitosis are negative for both reporters. **(E)** G0-Venus and G1-Cherry reporters in PC3 cells respond to serum starvation and re-stimulation as expected. G1 (Cherry-only) cells were isolated by FACS and cultured in serum free media for 3 days. By 3 days, 90% of cells become Venus/Cherry double positive demonstrating that nearly all cells retain the dual reporters. In parallel, double negative late S, G2/M cells were isolated by FACS and cultured in serum free media. By 3 days, 85% of cells become Venus/Cherry double positive, demonstrating that actively proliferating cells retain the dual reporters. Serum was then added back to G0 arrested cells, and within 2–3 days (days 5 and 7 of the entire timecourse) the distribution of G0, G1, S,G2/M cells returns to normal. **(F)** PC3 Venus/Cherry cells were cultured in a microfluidics chamber termed the “cell hotel” for single cell tracking and imaging of daughters. Examples of asynchronous G0/G1 decisions, as well as synchronous spontaneous G0 and synchronous G1 entry are observed in PC3 cells under full serum conditions. Orange arrows indicate cells entering G0 (G0-Venus, G1-Cherry double positive), red arrows indicate cells entering G1 (G1-Cherry only). **(G)** To measure heterogeneity of G0 in PC3 cells under full serum conditions, we quantified time spent in a double-positive Venus/Cherry state for 90 cells. We found G0 length to be highly heterogeneous, compared to the rest of the cell cycle timing for G1, S and G2/M. **(H)** We measured the length of the double Venus/Cherry positive G0 state for ∼50 PC3 and 3T3 cells under full serum conditions in the cell hotel. For PC3, we found that most cells transitioned to G1 by 14 h after the initial rise in G0-Venus fluorescence, with a small number of cells (27.5%) exhibiting longer G0-Venus fluorescence consistent with spontaneous quiescence. By contrast, for 3T3 cells we observed 64.4% of cells to exhibit spontaneous quiescence, a double-positive state lasting more than 14 h (dotted line). (*p* = 0.0005 by Mann-Whitney test.) **(I)** We also compared the frequency of asynchronous proliferation-quiescence decisions in PC3 vs 3T3 cells (*p* < 0.0001 by Mann-Whitney test) and **(G)** the length of the time difference until G1 entry between asynchronous daughters in PC3 and 3T3 cells. Lines show the mean and error bars are ±SEM from at least three independent experiments.

Cellular quiescence in prostate cancer is thought to contribute to tumor dormancy and issues with metastatic cancer recurrence. However, it is not well understood how and why prostate cancer cells enter and exit quiescence. We wondered whether spontaneous quiescence and asynchronous proliferation-quiescence decisions may, in part, underlie cell cycle heterogeneity in prostate cancer cells. To examine this, we transduced the mVenus-p27K^−^ G0 and mCherry-hCdt1 G1 reporters into PC3 cells. We initially selected pools of transduced cells expressing both reporters under reduced serum conditions by FACS. We found that sorted, pooled cells quickly lost expression of one or the other reporter after a limited number of passages. We therefore isolated clones and selected a clonal PC3 Venus-Cherry cell line, stably expressing both reporters at visible levels with normal cell cycle dynamics (i.e a cell doubling time similar to parental PC3). In this line, we readily observe double positive Venus/Cherry cells under normal full-serum culture conditions (Figure 2A) that are negative for EdU incorporation, negative for Ki-67 and both reporters are silent in cells that progress through the cell cycle into mitosis (Figures 2B–D). We also confirmed that the reporters exhibited the expected G0/G1 dynamics during serum withdrawal and serum re-addition in PC3 cells (Figure 2E).

We next attempted to track the reporter dynamics in PC3 cells with live cell imaging and found that these cancer cells were too motile to be tracked accurately for more than a few hours. We therefore used a microfluidic device we term the “cell hotel,” to capture one or a few cells and trap them in a chamber, to allow for manual tracking of individual cells and their daughters (Cheng et al., 2016). Each cell hotel slide allows simultaneous recording of up to 27 chambers under ×10 magnification. We confirmed that the PC3 Venus-Cherry cells in the cell hotel exhibited similar growth and cell doubling times as previously reported for PC3 cells in bulk cell culture. In addition we repeated measurements of 3T3 Venus/Cherry cells in the cell hotel for all comparisons to PC3 (Figures 2F–J).

Similar to the 3T3 cells, we observed a nearly invariant reporter progression of mVenus-p27K^−^ expression ∼2 h after cytokinesis, followed by mCherry-hCdt1expression approximately 2 h later. Cells that enter the cell cycle, degrade mVenus-p27K^−^ within approximately 4 h, followed by mCherry-hCdt1 degradation and ultimately cell division (Figure 2F). As in 3T3 cells, we observed 20% of cells with stabilized mVenus-p27K^−^ and mCherry-hCdt1 for 14 h and beyond, without division or evidence of S/G2/M entry for 20 h or more, suggestive of spontaneous G0 entry in PC3 cells (Figures 2G,H). Notably, spontaneous quiescence in PC3 cells tends to be more rare and shorter than in 3T3 cells (Figure 2H). This could reflect the important role for p53 signaling in spontaneous quiescence (Arora et al., 2017; Yang et al., 2017), as PC3 cells lack functional p53 (Carroll et al., 1993). We also observed evidence of asynchronous proliferation-quiescence decisions, with 30% of daughters making asynchronous G0/G1 decisions within 1–6 h of each other (Figures 2I,J). Interestingly, the asynchronous proliferation-quiescence decisions were also rarer and the difference in timing between asynchronous daughters was less dramatic in PC3 cells (Figure 2J).

### Tumor Dormancy Signals can Influence Quiescence and Asynchronous Proliferation-Quiescence Decisions

**FIGURE 3:**
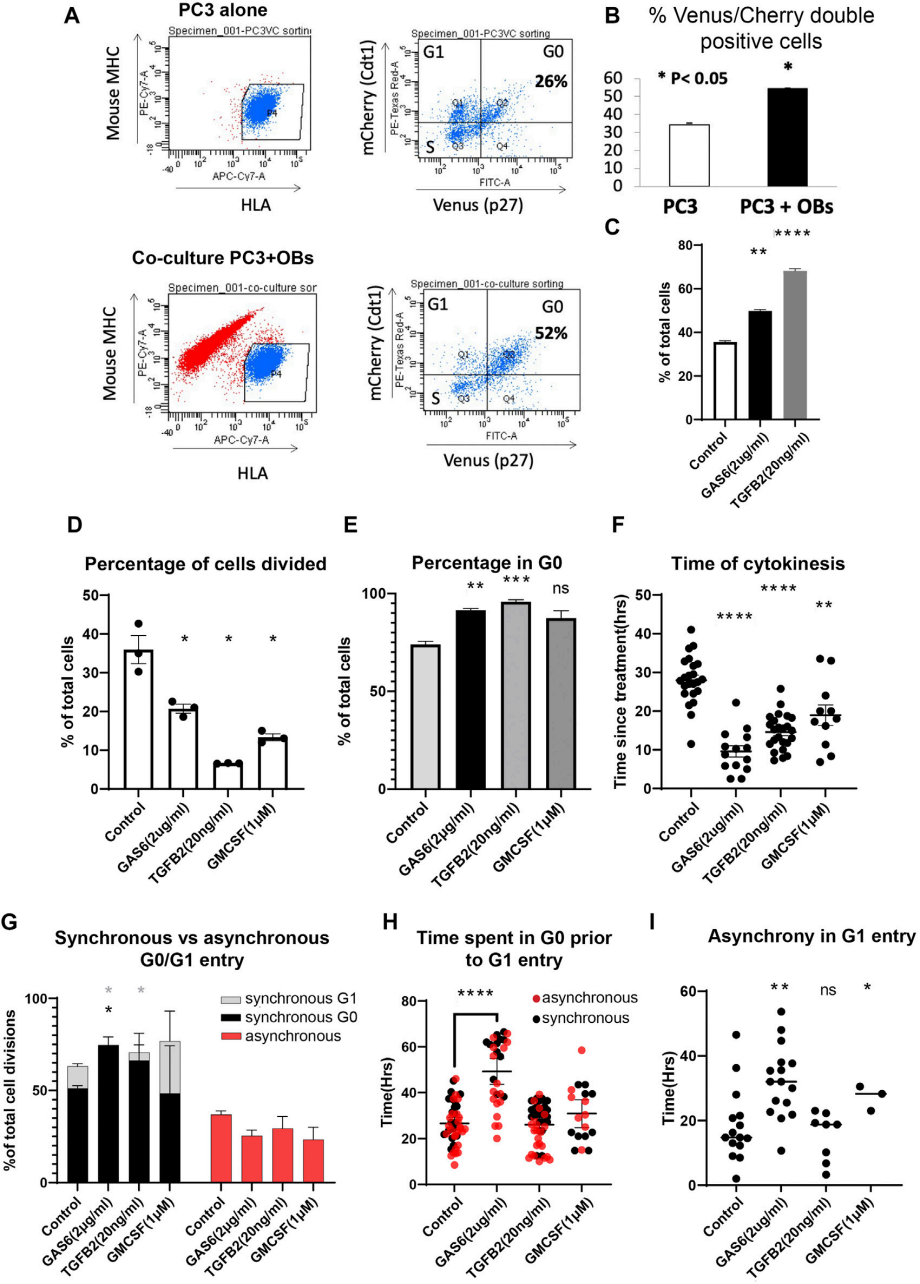
Tumor dormancy signals influence quiescence and asynchronous proliferation-quiescence decisions. **(A)** PC3 Venus/Cherry cells were either cultured alone or co-cultured with mouse osteoblasts, which were excluded from cell cycle analysis by negative human HLA staining and positive anti-mouse MHC staining. **(B)** PC3 co-culture with osteoblasts induced a significant increase in G0 cells under full serum conditions. **(C)** PC3 cells treated with Gas6 or TGFß2also exhibit a significant increase in G0 cells, measured by flow cytomtery. **(D)** G0-Venus reporter dynamics were tracked using the cell hotel for cells exposed to Gas 6 (*n* = 583), TGFß2 (*n* = 1,576) or GM-CSF (*n* = 330) or vehicle only controls (*n* = 336). Experiments were performed at least in triplicate. Gas6, TGFß2 and GM-CSF significantly decreased the percentage of cells that divide. **(E)** We quantified the percent of cells for each treatment that exhibited G0, defined as a Venus/Cherry double positive state for >14 h. **(F)** We tracked the timing of asynchronous cell divisions with Gas6, TGFß2 and GM-CSF treatment, and most divisions occurred significantly earlier followed by entry into quiescence. **(G)** Synchronous G0 entry, synchronous G1 entry and asynchronous G0/G1 entry was tracked for cell divisions in Gas 6 (*n* = 121), TGFß2 (*n* = 104), GM-CSF (*n* = 44) or vehicle only controls (*n* = 120). For Gas 6 and TGFß2 we observe a significant increase in synchronous G0 entry, while treatment with GM-CSF increased synchronous entry into G1. **(H)** To measure transient G0, we identified cells that spent more than 4 h in G0 prior to G1 entry and measured the length of their G0. Treatment with Gas6 significantly prolonged G0, even in cells that enter transient G0. Cells that enter G1 synchronously are in black, while asynchronous cells are in red. **(I)** For pairs of daughters that enter G1 asynchronously, we measured the difference in time for G1 entry. Gas6 significantly increased the time difference for asynchronous G1 entry. Lines or bars show the mean and error bars are ±SEM. All experiments were performed at least in triplicate and compared to controls with an unpaired *t*-test, * indicates *p* < 0.05, ** indicates *p* < 0.01, **** indicates *p* < 0.001.

Bone is a common site for prostate cancer metastasis and work from our group and others have shown that signals from osteoblasts can influence prostate cancer dormancy and PC3 cell cycle dynamics (Jung et al., 2016; Lee et al., 2016; Yumoto et al., 2016). Our previous work on PC3 cell cycle dynamics used the FUCCI cell cycle reporters, which could not distinguish between G0 and G1 arrest (Jung et al., 2016). We therefore examined whether PC3 Venus-Cherry cells co-cultured with osteoblasts increased entry into G0 quiescence. We found that co-culture with mouse MC3T3-E1 pre-osteoblasts under full serum conditions significantly increased the fraction of double positive PC3 Venus-Cherry cells consistent with increased entry into G0 (Figures 3A,B). We next examined whether Gas6 and TGFß2, signals from osteoblasts we have previously shown to induce a G0/G1 cell cycle arrest (Jung et al., 2016; Lee et al., 2016; Yumoto et al., 2016), induced entry into G0. Indeed, exposure to Gas6 or TGFß2 significantly increased the fraction of double positive PC3 Venus-Cherry cells after 48 h, suggesting the G0/G1 arrest we previously observed was indeed arrest in G0 (Figure 3C).

In the bone marrow, Gas6 and TGFß2 are thought to promote prostate cancer dormancy (Jung et al., 2012; Taichman et al., 2013; Ruppender et al., 2015; Yumoto et al., 2016) while GM-CSF promotes stem cell release from the bone marrow, which may provide cues for metastatic cancer cells in the bone marrow to exit from dormancy and proliferate (Dai et al., 2010). While some of the role for GM-CSF is thought to be due to indirect effects on the bone marrow stem cell niche, studies of GM-CSF directly added to cultured PC3 cells also show increased S-phase entry, proliferation and clonogenic growth consistent with exit from dormancy (Lang et al., 1994; Savarese et al., 1998). We therefore wanted to test whether GM-CSF impacted the proliferation-quiescence decision in PC3 cells and compare this to the effects of the dormancy associated signals Gas6 and TGFß2 on G0. We used the cell hotel to track cell cycle and mVenus-p27K^−^ and mCherry-hCdt1 dynamics in PC3 cells 2 h after the addition of Gas 6, TGFß2 or GM-CSF. In response to Gas6 and TGFß2 we observed a significant decrease in the number of cells undergoing divisions during the 72 h live imaging period, consistent with an increase in G0 entry (Figures 3D,E). Unexpectedly, we also observed a similar decrease in cell divisions for cells treated with GM-CSF, and an increase in Venus-Cherry double positive cells, suggesting GM-CSF also promotes G0 entry. For the fraction of cells undergoing divisions during the live imaging, we tracked when these cell divisions occur. We found that control cells asynchronously proliferate throughout the live imaging time course, while most cells treated with Gas6, TGFß2 or GM-CSF divide within the first 24 h of imaging (Figure 3F). These data suggest that cells uncommitted to the cell cycle either enter or remain in quiescence in response to Gas6, TGFß2 or GM-CSF. Cells that are past the restriction point when the treatment begins, and therefore committed to cycle, must make a subsequent proliferation-quiescence decision after mitosis that may be tipped toward quiescence. To confirm this, we examined the proliferation-quiescence choices made by pairs of daughters that divide under each treatment, broken down into: synchronous entry into G0, synchronous entry into G1 or asynchronous entry with one daughter entering G1 while the other remains in G0. For Gas6 and TGFß2 treated cells, we observed a significant increase in synchronous entry into G0 at the expense of synchronous entry into G1, with little impact on the proportion of cells that exhibit asynchronous proliferation-quiescence decisions (Figure 3G). This suggests that Gas6 and TGFß2 continue to promote quiescence in cells that are already committed to cycle after treatment addition. By contrast, GM-CSF treatment increased the proportion of divisions resulting in synchronous entry into G1, suggesting prolonged GM-CSF exposure may eventually promote cell cycle entry in a subset of the population (Figure 3G). Of note the pro-proliferative effects previously reported were seen in experiments performed on much longer timescales of at least 3–4 days (Lang et al., 1994; Savarese et al., 1998), suggesting the response to GM-CSF may be complex. Our data suggests GM-CSF treatment initially promotes quiescence entry in cells that are prior to the restriction point, but for a subset of cells past the restriction point it promotes their daughters to preferentially enter G1.

We next tracked pairs of daughters from the dividing cells under treatment and measured how long they spent in a Venus/Cherry double positive G0 state after mitosis prior to entering a Cherry-only G1 state. The goal was to determine whether each treatment also impacted the heterogeneity of transient quiescence, a feature that may initially promote tumor dormancy, but also lead to later recurrence. We found that only treatment with Gas6 impacted cells that entered transient quiescence, by significantly prolonging the time spent in G0 prior to the next G0-G1 transition (Figure 3H). This prolonged G0 occurred whether the pairs of daughters entered into G1 synchronously or asynchronously, and increased the differences in timing of G1 entry between pairs of asynchronous daughters (Figure 3I). This suggests that Gas6 promotes quiescence, but also promotes quiescence heterogeneity in cells that retain the ability to re-enter the cell cycle.

### Quiescent Cancer Cells are Enriched for Stem Cell Markers and Express High Levels of Hippo Pathway Signaling Components

**FIGURE 4:**
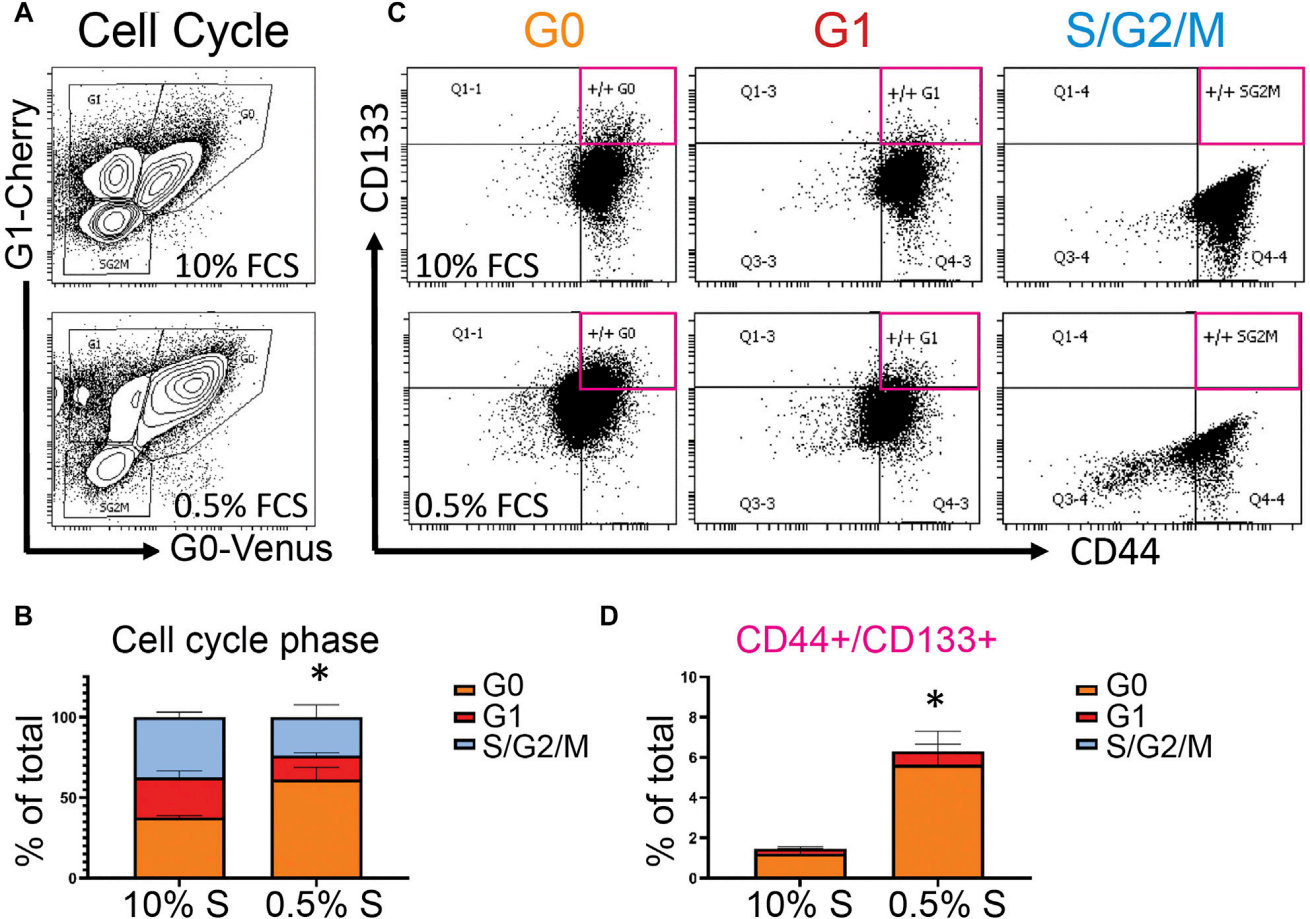
Quiescent prostate cancer cells are enriched for a subpopulation of cells that express potential cancer stem cell markers, **(A)** Flow cytometry plots of cell cycle phase of PC3 Venus-Cherry cells grown with 10% serum (FCS) or 0.5% serum (FCS) for 3 days **(B)** Quantification of the cell cycle phase data **(C)** Flow cytometry for CD133 and CD44 to assess cancer stem cell marker expression in each of the cell cycle populations. **(D)** CD133/44 double positive cells from each cell cycle phase group, quantified as a percentage of the total events in panel A. Quantified data in panels C and D represent mean ± SEM of three independent experiments. * represents *p* < 0.05 for the G0 population by Student’s *t*-test. Flow cytometry plots in panels A and B show a representative experiment.

**FIGURE 5:**
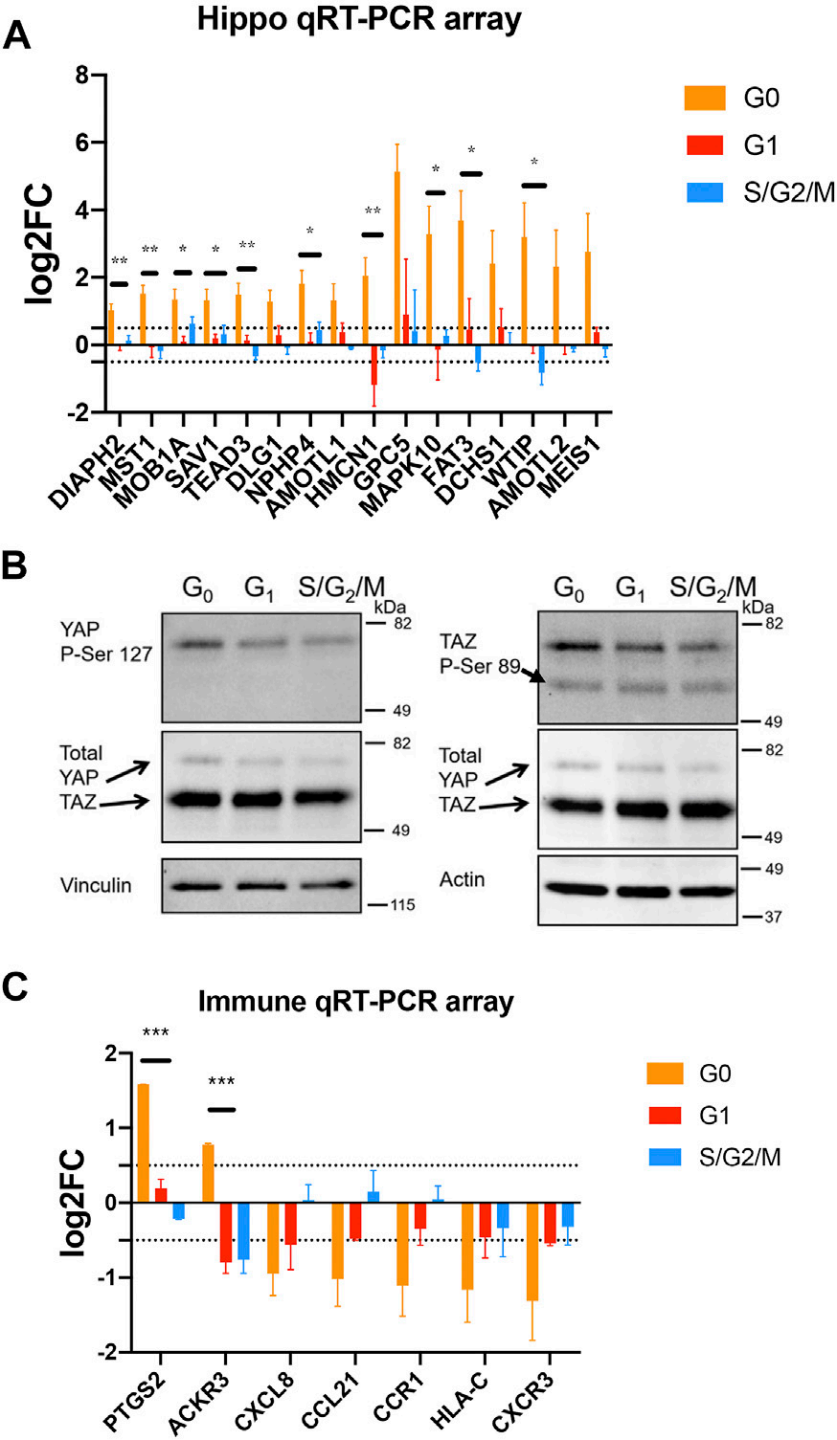
Quiescent prostate cancer cells exhibit altered expression of Hippo pathway components and immune-related genes. **(A, C)** PC3 Venus/Cherry cells were sorted into G0, G1 and S,G2/M fractions for gene expression analysis using Qiagen qRT-PCR arrays. Biological quadruplicates were run on the Hippo Signaling Pathway array and triplicates were run on the Qiagen Cancer Immunology qRT-PCR array. All changes in expression are normalized to asynchronous cells. Selected genes are shown here, the full dataset is shown in the Supplementary Figure S4B. **(A)** G0 cells show a consistent increase in transcripts for Hippo signaling pathway components including positive and negative regulators as well as feedback targets. **(B)** PC3 Venus/Cherry cells were sorted into G0, G1 and S,G2/M fractions for protein isolation and western blotting. G0 cells exhibit an increase in phosphorylated YAP consistent with active Hippo signaling, but little effect on TAZ (note TAZ protein encoded by WWTR1 gene). **(C)** G0 cells exhibit an altered immune expression profile while G1, S and G2/M cells exhibit few significant changes. CXCL8, CCL21, CCR1, HLA-C and CXCR3 were all significantly different from asynchronous controls by a 2-fold cutoff and *t*-tests with *p* < 0.02. For all gene expression data in A and C, G0 cells were also compared to G1 or S/G2/M cells by unpaired *t*-tests. * indicates *p* < 0.05, ** indicates *p* < 0.01, *** indicates *p* < 0.005.

Identifying molecular markers of quiescent cancer cells that could be assayed in patient samples is an attractive approach to identify those at risk for metastasis and recurrence. Toward this goal, we used the PC3 Venus-Cherry cells to isolate populations enriched for quiescent cancer cells by FACS, to examine their cell surface markers and gene expression changes. (Figures 4,5). We first assayed the prostate cancer stem cell markers CD133 and CD44 to determine whether increasing cellular quiescence could increase the fraction of CD133/CD44 double positive potential cancer stem cells (Jung et al., 2016). We cultured PC3 Venus/Cherry cells under normal 10% serum conditions, or reduced 0.5% serum conditions for 72 h. We confirmed an increase in the G0 population under reduced serum (Figure 4A, B) and compared the fraction of CD133/CD44 double positive cells in the G0, G1 and S/G2/M populations (Figure 4C). We observed the majority of the CD133/CD44 double positive cells to be in the G0 population, with a much smaller fraction in G1 and almost none in the S/G2/M population (Figures 4C,D). This suggests that signals in the tumor environment that increase the quiescent population in prostate cancer may also increase the number of potential cancer stem cells that could underlie recurrence.

We previously established a mouse xenograft model of prostate cancer bone metastasis using Du-145 cells, that recapitulates aspects of dormancy and recurrence (Cackowski et al., 2017). We attempted to use the PC3 Venus/Cherry cells in a similar xenograft model, but found that the cells quickly silenced the cell cycle reporters *in vivo*. We therefore used the xenograft model with Du-145 cells as a tool to compare gene expression profiles for cells in actively growing bone metastases as assessed by bioluminescence imaging (“involved”) vs bones without imaging detected metastases, but which still contained cancer cells that were fewer in number and presumably more slowly growing (“uninvolved”). Use of this approach of comparing cancer cells from high burden/involved vs low burden/uninvolved sites was previously used in a breast cancer model and showed that cancer cells from the uninvolved sites had more stem-like properties (Lawson et al., 2015). We isolated cells from mouse marrow under both conditions and performed bulk RNA-seq to compare global gene expression changes in the growing vs dormant state. Due to the small number of human cells recovered from uninvolved bones, we were only able to accurately assign differences in expression for 117 genes (Supplementary Figure S3). Nonetheless using DAVID and the KEGG database to perform pathway analysis of differentially expressed genes, we found an enrichment of genes involved in extracellular matrix interactions and the TGF-beta signaling pathway, factors known to impact prostate cancer dormancy and dormancy escape (White et al., 2006; Bragado et al., 2013; Ruppender et al., 2015). We noted that several of the genes falling into these enriched categories also interface with Hippo signaling, which is a key regulator of cell cycle exit (Zheng and Pan, 2019). We therefore decided to examine whether components of Hippo signaling may be altered in quiescent prostate cancer cells.

To examine differences in gene expression between G0, G1, and S/G2/M populations, we sorted populations and examined gene expression differences using a Hippo signaling Pathway qRT-PCR array (Figure 5A). In G0 cells, we noted a widespread increase in transcripts for Hippo signaling pathway components (e.g., DCHS1, FAT3, MST1, MOB1A, SAV1), transcriptional regulators (TEAD3, MEIS1) as well as targets (AMOTL1, AMOTL2) (Wang et al., 2018). The increased expression of some transcriptional targets of Hippo signaling is surprising, since these cells are in G0 and therefore would be expected to have Hippo signaling on. Hippo signaling acts via phosphorylation to suppress the activity of the downstream transcriptional effectors YAP1 and TAZ (encoded by the WWTR1 gene in humans) (Zheng and Pan, 2019). We therefore examined whether YAP and TAZ are suppressed via phosphorylation in G0 cells through active Hippo signaling. We performed westerns for the inactivating phosphorylations on YAP1 and TAZ in G0, G1 and S/G2/M sorted cells (Figure 5B) and found a small increase in phosphorylated YAP, but no effect on TAZ. We hypothesize the increased expression of Hippo pathway components may poise quiescent cells to re-enter the cell cycle upon receipt of a dormancy escape signal, but that during G0, active Hippo signaling restrains YAP transcriptional activity through inhibitory phosphorylation. (Figure 5 and Supplementary Figure S4).

Hippo signaling in cancer has been associated with restraining proliferation (Zheng and Pan, 2019), but also has been shown to alter immune response, with active Hippo signaling suppressing tumor immunogenicity (Moroishi et al., 2016; Yamauchi and Moroishi, 2019). We therefore next examined whether G0 cells exhibited alterations in expression of immune response-associated signals. Using the Qiagen Cancer Immunology array on sorted G0, G1 and S/G2/M PC3 cells vs the mixed population as a reference, we observed a moderate but widespread decrease in the expression of immune-related genes including signals known to target cancer cells for host immune destruction, such as CXCR3, CXCL8, and HLA-C (Figure 5C) in G0 cells. This suggests that spontaneously quiescent cancer cells may exhibit altered immunoreactivity. Interestingly, one pro-inflammatory gene, PTGS2 (Cox-2), was strongly upregulated in G0 cells (Figure 5C), consistent with the previous work showing this target to be de-repressed when upstream Hippo signaling is active and YAP/TAZ are suppressed by phosphorylation (Zhang et al., 2018). Taken together, our results suggest the inherent cell cycle heterogeneity of metastatic prostate cancer includes a fraction of spontaneously quiescent cells that are enriched for cells expressing cancer stem cell markers and exhibit gene expression changes consistent with a state poised to re-enter the cell cycle, but potentially less visible to the host immune system.

## Discussion

Several cancers contain heterogeneous populations with varying levels of proliferation (Davis et al., 2019). Some studies suggest that quiescent tumor cells contribute to drug-resistance, by providing a population of non-cycling cells that survive cytotoxic chemotherapy (De Angelis et al., 2019; Talukdar et al., 2019; Hen and Barkan, 2020). Understanding the molecular basis of proliferative heterogeneity therefore may assist in developing better therapeutic approaches for cancer. Here, we show that untransformed 3T3 cells and PC3 prostate cancer cells show spontaneous quiescence and heterogeneous G0 lengths under pro-proliferative culture conditions. We propose that spontaneous quiescence may be related to quiescence in cancer, since quiescent cancer cells must leave the cell cycle in the presence of pro-proliferative growth factor and oncogenic signaling.

Spontaneous quiescence has been shown to underlie clonal cell cycle heterogeneity (Overton et al., 2014) and may in part underlie cell cycle heterogeneity in tumors. Here we show an additional mechanism to create heterogeneity, asynchronous G0/G1 decisions, where one daughter from a mitotic event remains in G0, while the other enters G1. These asynchronous decisions are somewhat surprising, since recent work has suggested the signals that influence the proliferation-quiescence decision are integrated over the previous cell cycle phases prior to mitosis and therefore would be expected to be inherited equally in daughters after mitosis (Yang et al., 2017; Min et al., 2020). The asynchrony in asynchronous PC3 cell divisions is often only a few hours but can extend to over 20 h or more in the presence of the dormancy inducing factor, Gas6 (Figure 3I). Small differences in asynchronous pairs of daughters may possibly be explained by fluctuations resulting in unequal protein and transcript inheritance at mitosis. However, this is a less satisfying hypothesis for differences in G0 exit between asynchronous daughters longer than 10 h. Previous work in MCF7 breast and HCT116 colon cancer cells has shown a population of dormant cells resulting from asymmetric Akt signaling after cell divisions (Dey-Guha et al., 2011). In this example, about 1% of cell divisions exhibit asymmetry, resulting in a daughter with low Akt signaling. Importantly, elimination of Akt prevented proliferative heterogeneity in these lines in cell culture (Dey-Guha et al., 2015), and inhibition of asymmetric Akt signaling reduced tumor recurrence after treatment in a xenograft model (Alves et al., 2018). It is worth noting that PC3 cells lack functional PTEN (Huang et al., 2001; Dubrovska et al., 2009) and therefore would be expected to have higher endogenous Akt signaling that suppresses some degree of asymmetry. In addition, Gas6 and other TYRO3/AXL/MERTK ligands signal in part through Akt, and therefore may also impact Akt asymmetry (Cosemans et al., 2010; Kasikara et al., 2017). Inhibition of Akt signaling can lead to up regulation of p27 in PC3 cells (Van Duijn and Trapman, 2006), while over expression of p27 can also inhibit Akt signaling (Chen et al., 2009). Further work will be needed to determine if asymmetric Akt signaling may be a cause or consequence of asynchronous proliferation-quiescence decisions in prostate cancer.

The relationship of dormancy and cellular quiescence remains unclear. Here we show that dormancy-associated signals in prostate cancer, Gas6 and TGFß2, rapidly within a few hours, induce quiescence entry in prostate cancer cells after mitosis. This is in part because these signals tip the balance of proliferation-quiescence decisions in favor of synchronous G0 entry. By contrast, a presumed pro-proliferative signal for PC3 cells, GM-CSF tips the balance in favor of synchronous divisions into G1 for the cells that divide after initial exposure. Thus, although GM-CSF promotes G0 entry initially, sustained signaling may promote cell cycle re-entry in the longer term. Gas6 also has a complex effect on the proliferation-quiescence decision. In addition to promoting G0 in cells that are uncommitted to the cell cycle, Gas6 also prolongs G0 in cells that retain the ability to eventually re-enter the cell cycle. This suggests that the quiescence response to Gas 6 is not an all or nothing response and it can be graded, resulting in varying lengths of G0 to promote quiescence heterogeneity. While none of the signals we tested significantly altered the frequency of asynchronous cell cycle entry in pairs of daughters after mitosis, Gas6 significantly increased the asynchrony in G0 exit and G1 entry. We suggest this could be another source of quiescence heterogeneity in cancer.

Understanding the gene expression changes in dormant cancer cells will be essential to understanding their biology, but will also be useful tools as molecular markers for identifying them in patient samples. Here we show that quiescent PC3 cells are enriched for prostate cancer stem cell markers CD133 and 44 and that driving quiescence entry through serum starvation significantly increases the population of CD133/44 double positive cells in the population. Quiescent prostate cancer cells also exhibit increased expression of some Hippo pathway components, while the Hippo pathway remains on to restrain cell cycle entry. This finding in cell culture was also supported by our gene expression analysis in an *in vivo* xenograft model for prostate cancer tumor dormancy (Supplementary Figure S3). Interestingly, this is correlated with suppressed levels of mRNA for immune targeting factors, and may suggest a mechanism by which quiescent cancer cells evade host immune attack. Whether there is a direct or indirect relationship between the Hippo signaling status and expression of immune targets in quiescent cells remains to be examined.

## Materials and Methods

### Cells and Cell Culture

The mouse embryonic fibroblast 3T3 cell line containing the G0 and G1 cell cycle reporters were kindly provided by Dr. Toshihiko Oki (University of Tokyo). These cells were grown in Dulbecco’s modified Eagle’s medium (DMEM; Gibco) supplemented with 10% fetal bovine serum (FBS) and 1% penicillin-streptomycin. Serum levels were reduced as indicated in the figures and text for serum starvation experiments. PC3 prostate cancer cells were cultured in RPMI medium with 10% serum and 1% penicillin-streptomycin and transduced with the G0 and G1 cell cycle reporters as previously described (Takahashi et al., 2019).

### Live Cell Imaging

NIH/3T3 cells were cultured at low density (to avoid contact inhibition) on 12-well plates in phenol red-free DMEM/10%FBS or 1%DMEM. Experiments in Figure 1 (and Supplementary Figure S1) were performed using an EVOS FL cell imaging system with a ×20 objective lens or an IncuCyte Zoom at 37°C, 5% CO_2_. The imaging intervals were 20–30 min.

For experiments using the “Cell Hotel” (Figures 2, 3), 10,000 PC3 cells in RPMI medium with 10% serum were loaded into the inlet of the microfluidic chamber. The chamber loading was monitored until most of the chambers were occupied with single cells (∼5 mins). Remaining cells were then removed from the inlet and the outlet and replenished with fresh media. Imaging was performed using a Leica DMI 6000 with a Tokai Hit stage-top environmental chamber at 37°C, 5% CO_2_. TGFß2, Gas6 and GM-CSF (R&D systems) were reconstituted according to the manufacturer’s guidelines (R&D systems). For Gas6, TGFß2 and GM-CSF treatments, 10,000 PC3 cells were mixed with media containing the ligand (2 μg/ml for Gas6, 20 ng/ml for TGFß2 and 1 μM for GM-CSF) and introduced into the chamber. Cells were then incubated in media with the indicated ligand for 2 h of pre-equilibration prior to imaging every 30 min.

### Fabrication of the Microfluidic Device for Single-Cell Tracking

The microfluidic device used for single-cell tracking was developed in our previous work (Cheng et al., 2016). The device was built by bonding a PDMS (Polydimethylsiloxane, Sylgard 184, Dow Corning) layer with microfluidic patterns to a glass slide. The PDMS layer was formed by standard soft lithography. The SU-8 mold used for soft-lithography was created by a 3-layer photolithography process with 10 μm, 40 μm, and 100 µm thick SU-8 (Microchem) following the manufacturer’s protocol. PDMS was prepared by mixing with 10 (elastomer): 1(curing agent) (w/w) ratio, poured on SU-8 molds, and cured at 100°C overnight. Inlet and outlet holes are created by biopsy punch cutting. The PDMS with microfluidic channel structures and the glass slide were treated using oxygen plasma (80 W for 60 s) and bonded. The devices after bonding were heated at 80°C for an hour to ensure bonding quality. The microfluidic chips were sanitized using UV radiation and primed using a either a Collagen solution (1.45 ml Collagen (Collagen Type 1, 354,236, BD Biosciences) or Fibronectin solution, 0.1 ml acetic acid in 50 ml DI Water) overnight before use.

### Flow Cytometry Analysis and FACS

For cell sorting and flow cytometry assays in Figures 2–5, cells were cultured in RPMI supplemented with either 10% FBS or 1% as indicated and subpopulations were sorted according to the intensity of their fluorescent reporters, using a BD FACS Aria II system. Cells were sorted into SDS-PAGE loading buffer or RLT (Qiagen) for immediate protein extraction or RNA isolation. A minimum of ∼10^5^ cells were collected for each experiment. Antibodies used for PC3 isolation from osteoblasts co-culture in Figure 2 were APC/Cy7 anti-human HLA A,B,C antibody (Biolegend #311426).

For Figure 4, we assessed PC3 cells for dual positivity for CD44 and CD133 as we previously described (Shiozawa et al., 2016). PC3 cells were seeded at 10^5^ cells per well of six well plates in RPMI with 1% penicillin/streptomycin and either 10% or 0.5% serum, then cultured for 3 days. Both adherent and floating cells were analyzed for flow cytometry using a four laser BD LSR II instrument and FACSDiva™ software. We plotted G0-Venus vs G1-Cherry from the single, viable (DAPI negative) population and drilled down from each cell cycle phase group (G0, G1, or S/G/M) to analyze the percent CD133+/CD44 + cells from each population. Antibodies were PE-vio770 conjugated CD133/1, clone AC133 (Miltenyi Biotech #130–113–672) diluted 1:50 and APC conjugated CD44 (BD #559942) diluted 1:5.

### Western Blotting

Cleared cell lysates in SDS loading buffers were separated on 4–20% SDS PAGE gels under reducing conditions and transferred to PVDF membranes. Membranes were blocked with 5% milk in TBST and probed with primary antibodies diluted 1:1,000 in 5% BSA TBST; YAP1 phospho-serine 127 (Cell Signaling Technology #4911) and TAZ phospho-serine 89 (Cell Signaling Technology #59971). The secondary antibody was Cell Signaling #7074 diluted 1:1,000 in 1% milk TBST. Blots were developed in Pierce Supersignal Pico ECL substrate and visualized with a Biorad Image Doc Touch system. The membranes were subsequently stripped and reprobed for total YAP1 (CST #14074), total TAZ (CST #83669), beta actin (CST #4970), or vinculin (CST #13901) as indicated.

### qRT-PCR Arrays

PC3 Venus/Cherry cells were seeded at 10^5^ cells per dish in 10 cm dishes and cultured for 3 days in RPMI media with 1% FCS. Cells were seeded on different days for biologic triplicate or quadruplicate samples. After 3 days of culture, cells were released by tripysinization, stained with DAPI for viability and sorted by FACS into either the total (mixed) viable cell population, G0, G1, or S/G2/M phases using the Venus/Cherry markers. 10^5^ viable cell events for each population were collected directly into Qiagen RLT buffer containing β-mercaptoethanol. Total RNA was isolated with Qiagen RNeasy kits. The samples were analyzed with the Human Hippo Signaling RT2 Profiler PCR array (Qiagen #PAHS-172ZA) or Human Cancer Inflammation and Immunity Crosstalk array (Qiagen #PAHS-181ZA) using the recommended cDNA synthesis and PCR reagents. Data are presented as biologic quadruplicate or triplicate samples of expression relative to the total viable population sample. Visualization and hierarchical clustering was prepared with Morpheus software (Broad Institute).

Additional Methods and details for ATCQ and Supplemental Figures are included in the Supplemental Data file.

## Data Availability

The original contributions presented in the study are included in the article/Supplementary Material, further inquiries can be directed to the corresponding authors.
