# Supplementary figures and images for: Monitoring Spontaneous Quiescence and Asynchronous Proliferation-Quiescence Decisions in Prostate Cancer Cells
Source: Front Cell Dev Biol. 2021 Dec 10;9:728663. doi: 10.3389/fcell.2021.728663 (PMC8703172; doi:10.3389/fcell.2021.728663)

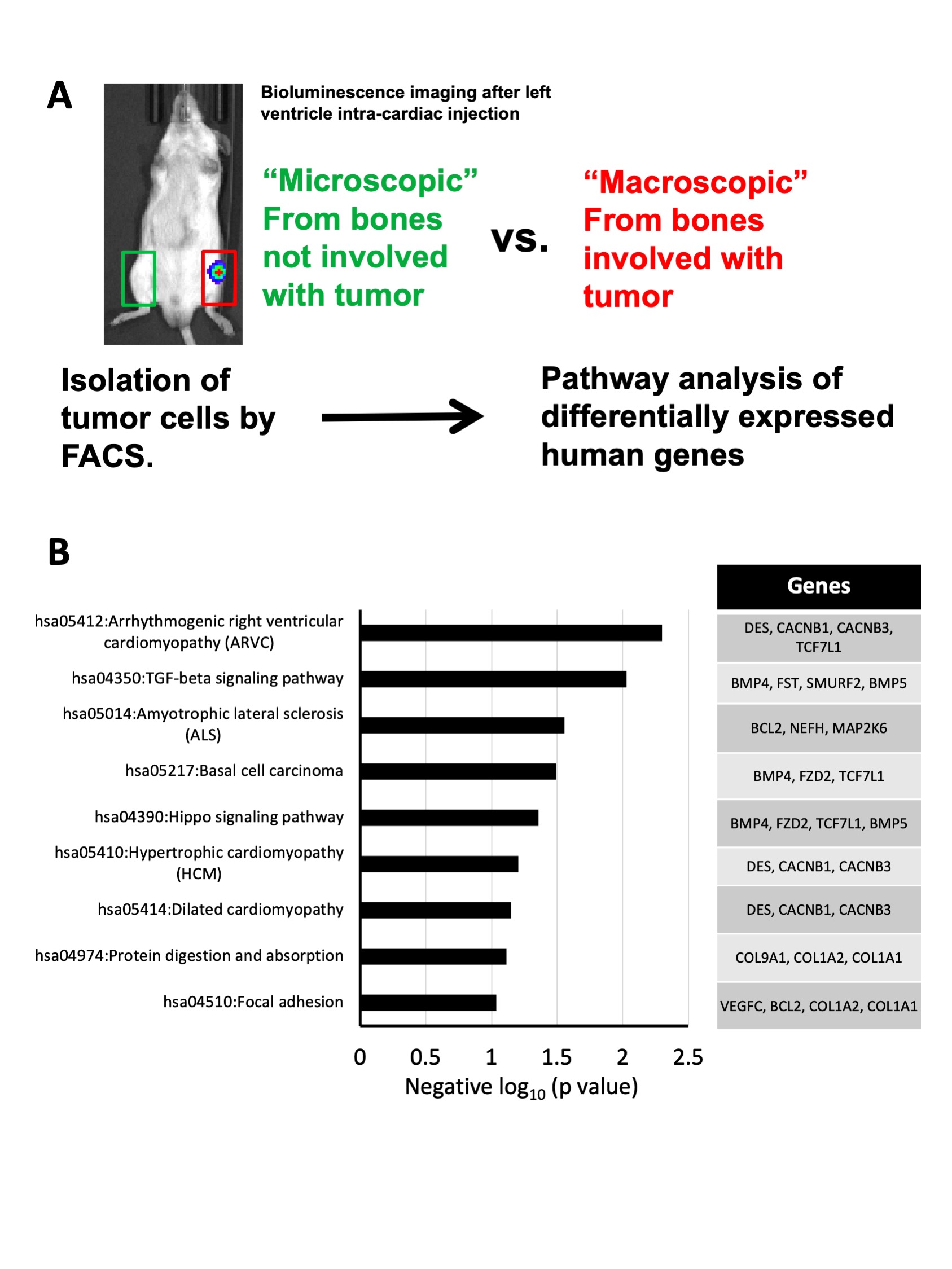

Supplement: Supplementary file 2 [file Image3.JPEG]

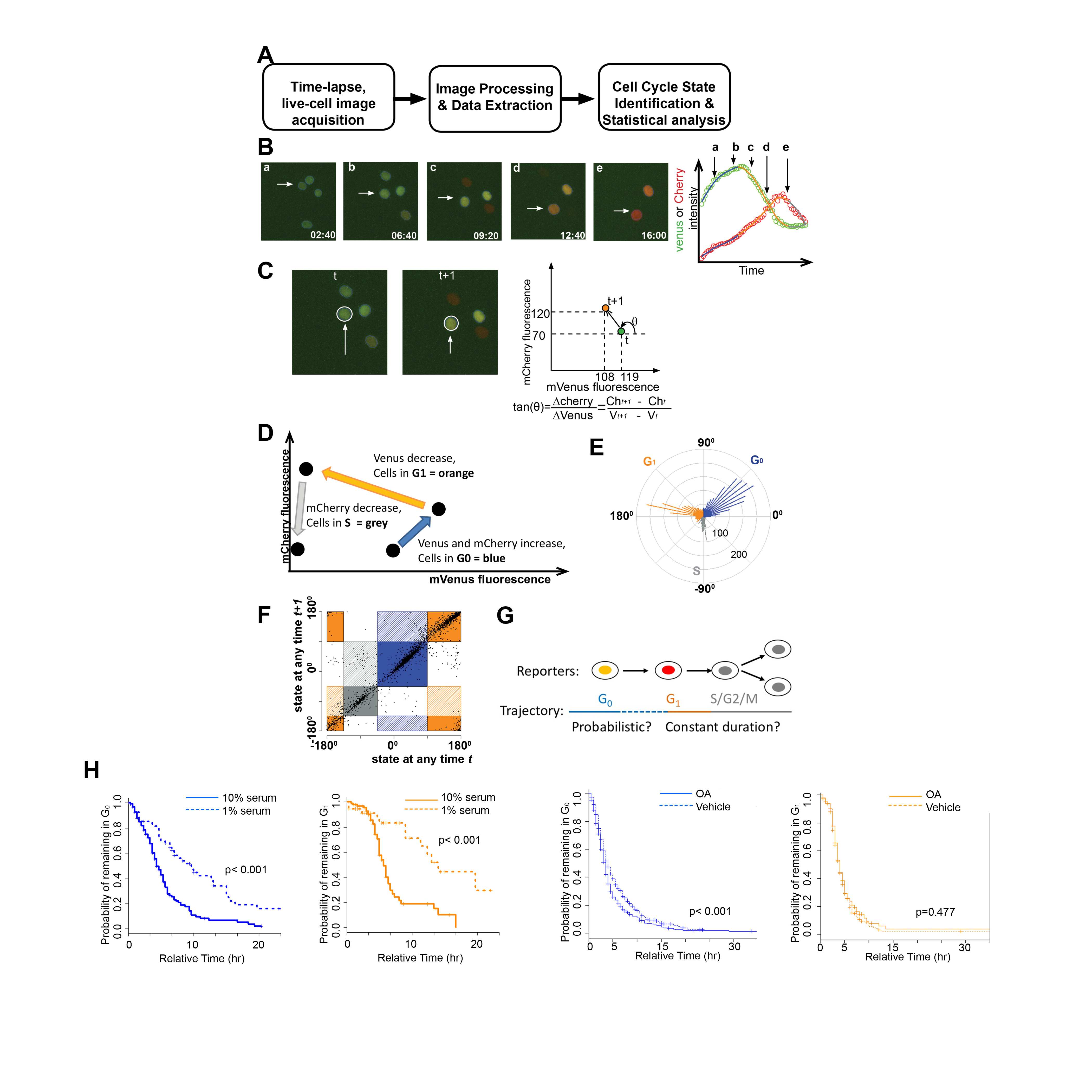

Supplement: Supplementary file 4 [file Image1.JPEG]

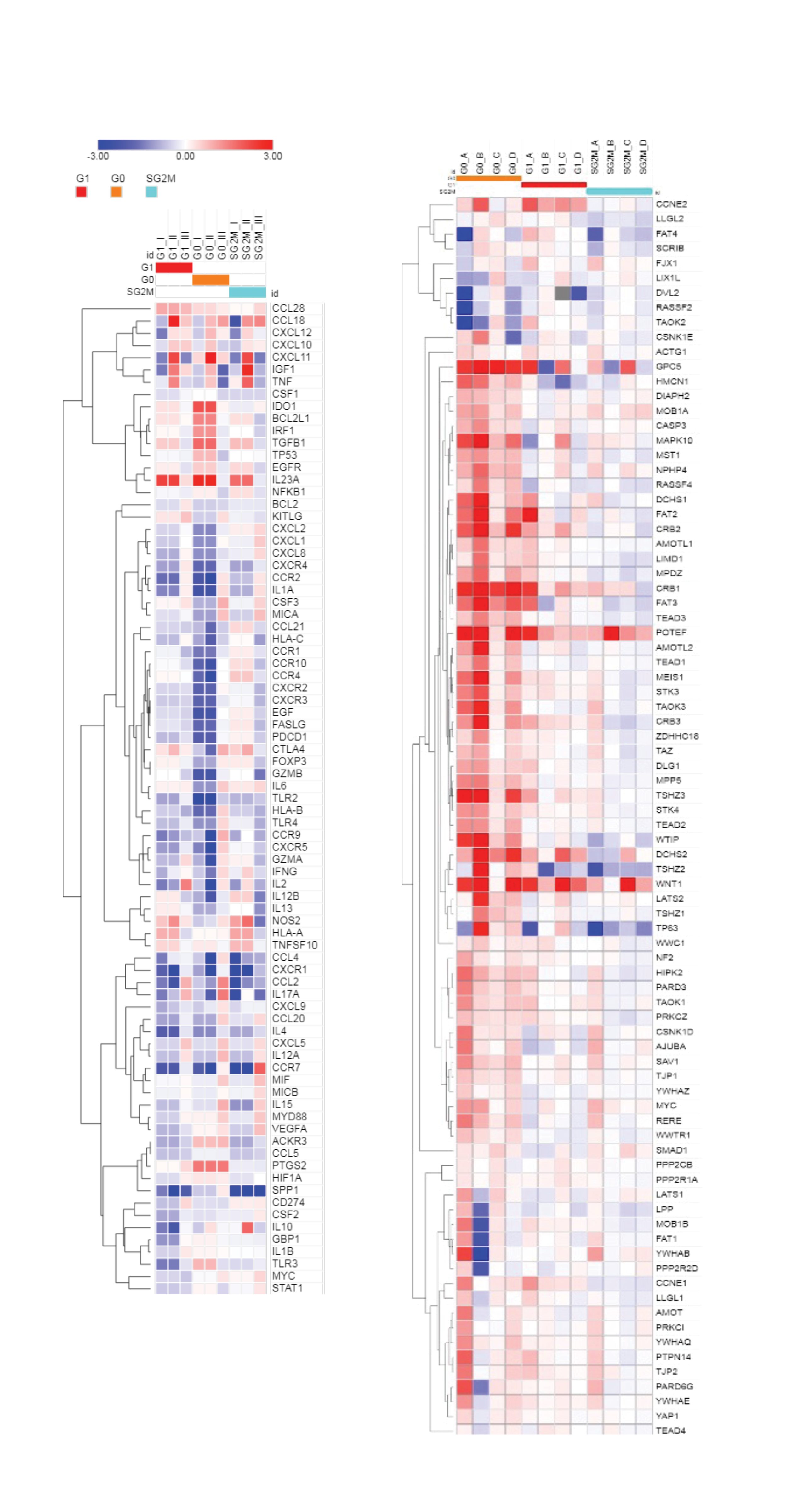

Supplement: Supplementary file 5 [file Image4.JPEG]

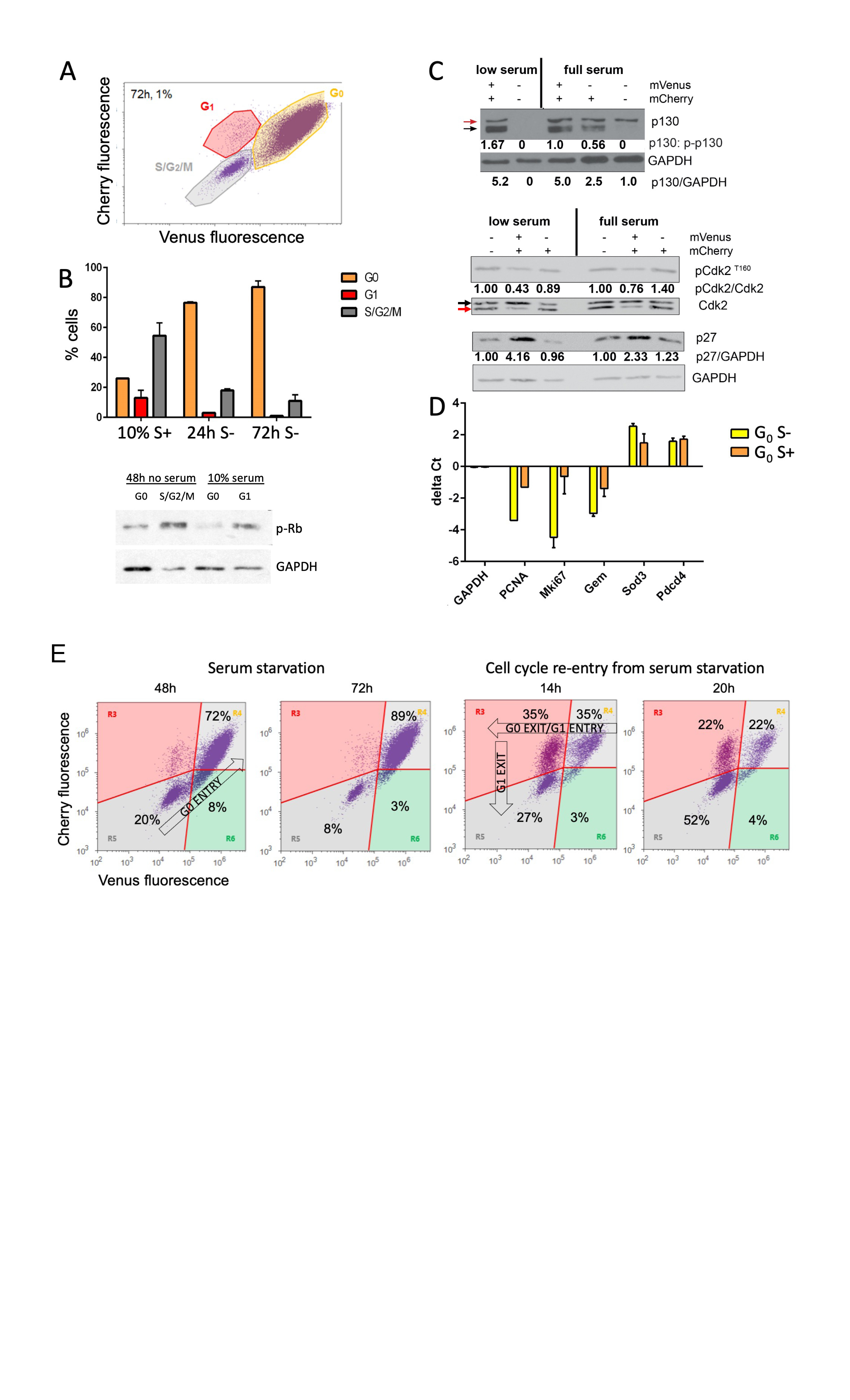

Supplement: Supplementary file 6 [file Image2.JPEG]
