# Supplementary material for: Monitoring Spontaneous Quiescence and Asynchronous Proliferation-Quiescence Decisions in Prostate Cancer Cells
Source: Front Cell Dev Biol. 2021 Dec 10;9:728663. doi: 10.3389/fcell.2021.728663 (PMC8703172; doi:10.3389/fcell.2021.728663)
Supplement: Supplementary file 8 [file DataSheet1.pdf]

Supplemental Methods

Appendix 1. Automated Tracking of Cellular Quiescence (ATCQ) Platform:

To monitor and quantitatively measure the dynamic transitions of cell cycle states—from cytokinesis to S phase entry, we developed the Automated Temporal Tracking of Quiescence (ATCQ) analysis platform that includes a computational framework for automated cell segmentation (identification of individual cells in an image), tracking, cell cycle state identification, and quantification from movies (Supp Fig.1). The cell segmentation and tracking allows us to record the fluorescent reporter intensity changes within individual cells in real-time imaging, without the aid of a constitutive nuclear marker. The single-cell fluorescence changes over time, in turn, are used to obtain cell cycle state identification (G0, G1, or early S phase) and quantification, which allows us to examine the kinetics of the proliferation-quiescence transition. The single-cell trace of fluorescent reporters, mVenus-p27K$^-$ and mCherry-hCdt1, graphed by ATCQ is consistent with what we manually observed in movies (Supp Fig.1).

We used the temporal changes in the fluorescence intensity readings of both reporters to create a classification that automatically assigned cell cycle phases for single cells (Supp Fig.1D). To quantitatively assess the relative change of two fluorescent reporters, we converted the relative change of the intensity readings from two adjacent time frames, t and t+1, into radian values (Supp Fig.1C). That is, the vector from time t to time t+1 on a plot of mCherry vs. mVenus fluorescence intensities is calculated into radian values over the range from –180° to 180°. A radial histogram of all recorded fluorescent vectors therefore appropriately reflects three primary clusters of cellular behaviors, corresponding to G0 entry, G0 exit or G1 entry, and G1 exit or early S entry. For example, a radian of -90° indicates cells exit G1 and enter into early S phase as mCherry signal decreases, while a radian of –180° indicates cells exit G0 and enter G1 as mVenus signal decreases while mCherry remains high.

This allows us to quantitatively visualize three distinct cell cycle behaviors at the proliferation-quiescence transition from the analysis of over 400 single-cell traces. The radial histogram exhibits the frequency and variability of three cell cycle behaviors in a population of asynchronously growing cells under normal, unperturbed conditions. The length of the bars

depicts the frequency of individual cells with two fluorescent reporters exhibiting a specific behavior assigned to each radian. The clustering of cells into three distinct states is marked by the local minima of the frequency in radial histogram. Therefore, a given cell at any internal time is associated with state 1: G0, state 2: G1, or state 3: S phase. To facilitate the visualization of this, cells in G0 state are labeled in blue, while cells that exit G0 and progress into G1 phase are labeled in orange. When cells enter S phase, they are labeled in grey (Supp Fig.1D-F).

To assess the accuracy of our approach, we generated a plot to show all the cell-level radian assignment values for temporally adjacent movie frames. In this plot, all the radian values defining cell state can be compared among all cells in any movie frame *t* and the next frame *t+1* (Supp. Fig. 1F). Each dot represents a single cell with the radian vectors drawn from adjacent time frames. Therefore, cells remaining in the same state within a time interval lie on the diagonal of the plot, while cells that transit between two states scatter above or below the diagonal. We considered cells to be correctly assigned if they either remained in their current state in the next frame, or cycle forward in the next frame. Our approach had a very high correct assignment rate, with 97-98% of frame intervals assigned with the correct cell-cycle transition order. This validates the feasibility of using ATCQ to investigate cell cycle dynamics. Furthermore, because ATCQ uses the relative changes in intensity of the two cell cycle reporters over time to assign a cell cycle state, correct assignments are not affected by cell to cell variation in fluorescence intensity.

Validating ATCQ

Single-cell, real-time imaging produces large amounts of data, which is advantageous when assessing heterogeneous cell dynamics in a population. In order to quantify the proliferation-quiescence decision with the large amount of imaging data, we used Kaplan-Meier (K-M) curves to estimate the time cells spent in a certain state (Supp. Fig.1H). The Kaplan-Meier estimator is a common statistical analysis tool to estimate clinical study survival functions that allows for individuals to be censored when they leave the study population (Kaplan and Meier, 1958). For our application, we took an advantage of it to estimate the probability of individual cells remaining in each state as a function of time. In the K-M curve, each step-down means cells exit that certain state. To compare time-in-states between treatment conditions, the time each cell spends in each state is first recorded, as well as whether a transition to the next state was

observed. If the completion of the state was not observed, that cell was censored. Censorship would occur for example, if a cell underwent apoptosis or if it moved out of the observation frame.

To test whether ATCQ is able to capture impacts of environmental stimuli on the proliferation-quiescence decision, we performed a serum starvation to promote G0 entry. As expected, cells under serum deprivation exhibit a significant difference in G0 (blue) but not S phase. The increase in prolonged G0 upon serum starvation, is evident from the slower decline of the curve compared to the full serum conditions. However, cells also tended to have a longer G1 phase under serum starvation compared to full serum conditions. This result was initially unexpected, but may be consistent with a recent study showing that cells in G1phase can delay DNA replication in response to serum starvation (Cappell et al., 2016). ATCQ thus reveals dynamics of the proliferation-quiescence decision consistent with single-cell studies of others, and will facilitate further studies of this transition under various environmental perturbations (Supp. Fig.1H).

Previously, we revealed a role for PP2A complex in restricting Cdk2 activity after mitosis to modulate the proliferation-quiescence decision in tissue development (Sun and Buttitta, 2015). We found that compromising PP2A activity caused 10% cells to fail to enter a developmentally regulated G0 state at the proper time. To examine whether PP2A affects G0 in cell culture, we performed a short-term (1h) treatment with the pan-PP2A inhibitor Okadaic Acid (OA) prior to imaging. As expected, analysis of the survival distributions showed that OA treatment results in a modest but statistically significant decrease in the median time in G0 (160 minutes vs. 140 minutes, $p < 0.001$). By contrast, no significant difference was observed for the time spent in the other two states (G1 and early S phase) (Supp Fig.I). Importantly the modest decrease observed in the G0 state is consistent with that expected if approximately 10% of cells are affected as previously shown (Naetar et al., 2014;Sun and Buttitta, 2015). This suggests that PP2A plays a role in promoting entry and maintenance of the G0 state in a fraction of cells and that ATCQ system can be sensitive enough to quantify the effects of perturbations that affect the proliferation-quiescence decision in a small fraction of cells.

Detailed Methods for ATCQ:

Live cell imaging: Cells were cultured at low density (to avoid contact inhibition) on 12-well plates in phenol red-free DMEM/10%FBS or 1%DMEM. Cells were imaged using an EVOS FL cell imaging system with a 20X objective lens or an IncuCyte Zoom at 37°C, 5% CO_2. The imaging intervals were 20-30 minutes. For PP2A inhibition, cells were exposed to Okadaic Acid (OA) at 50nM or DMSO (control) for 30 minutes. Media containing OA or DMSO was then removed, and fresh complete medium was added. Cells were imaged one hour later.

Image processing & analysis: Individual cell identification (segmentation) and tracking were performed using a custom MATLAB program that automates these processes as described below. The program interface provides interactive adjustment of the parameters used at each step in the segmentation process, allowing the user to tailor the segmentation process to accommodate the characteristics of each image under analysis. Then, cell tracking is done with the Simple Tracker tool available on the MATLAB File Exchange. This tool uses the Hungarian algorithm to link cells from frame to frame into connected tracks using user-entered parameters for maximum movement distance allowed between frames and maximum gap in frames allowed between occurrences of the same cell track. Cells were tracked for the entire time they were visible in the field of view. A subset of tracks were verified by hand, to be sure that one track represented the same cell for the duration of the track. The output of this software is an Excel file containing the identified cells, the frame number(s) in which they appear, the size, approximate radius, and cell center location in each frame, and the average and maximum intensity values in the cell on both the red and green channels in each frame, as well as the segmentation settings used to obtain these results.

ATCQ Statistical analysis

Fluorescence Intensity smoothing: Analysis was restricted to cells tracked over at least 3 video frames. The mCherry and mVenus fluorescence signals were smoothed using a nearest neighbor proportion localized polynomial approach. The localized polynomial nearest neighbor value parameter was chosen using an Akaike Information Criteria (Akaike, 1974) based optimization with leave one out cross-validation. Calculations were performed using the Locfit package (2) in the software program R 3.0.1(R Core Team, 2014).

Transformation to survival time data: For each cell, the cell cycle state over each pair of time points was assigned as described as before. For each cell, the aggregated time spent in each state was calculated. The event class for the last time point in each state was assigned as either "correct cycle" or "censored". Cells that were not assigned to progress forward in the cell cycle were censored from the analysis.

Statistical calculations: Associations between experimental time and overall fluorescence intensity were calculated via the Pearson's correlation coefficient and associated test (Tukey, 1949). Survival curves were generated via the Kaplan-Meier method and compared using the log-rank test. Survival time statistics were calculated using the survival package in R 3.0.1 (https://github.com/therneau/survival). Repeated measures linear regression was performed using generalized least squares regression using the nlme package with individual cells as the within-factor level (Cox, 1972;Pinheiro et al., 2017).

Appendix 2: Du-145 Xenograft RNAseq comparison

Tumor cells were isolated by FACS from the CB17 SCID mice injected in the left cardiac ventricle with luciferase and GFP labeled Du145 PCa cells expressing a control shRNA construct from our previous report (Cackowski et al., 2017). Briefly, the presence of tumor was determined by bioluminescence imaging. Tibiae, femorae, pelvic and lumbar vertebrae bones with or without detectable tumor (involved vs. uninvolved) were dissected and crushed with a mortal and pestle to release marrow cells. Mouse cells were depleted with a mouse cell depletion kit (Miltenyi #130-104-694). Cells were incubated with an APC/Cy7 anti-human HLA A,B,C antibody (Biolegend #311426) and a PerCP/Cy5.5 conjugated anti-mouse hematopoietic lineage cocktail antibodies (BD Biosciences #561317) and DAPI. Cancer cells were isolated as single / viable / lineage negative / HLA^{+} cells and collected in Qiagen RLT buffer containing β-mercaptoethanol. Total RNA was isolated using the Qiagen RNeasy micro kit (#74004). After RNA quality assessment with an Agilent 2100 Bioanalyzer, we selected paired samples from two mice for further analysis. Libraries were prepared using the Takara Clontech SMART-seq V4 Ultra Low kit for single cell analysis – which is also appropriate for small numbers of bulk isolated cells. We then acquired approximately 40 million 50 nucleotide paired end reads per sample on an Illumina Hi-Seq 4000. Tuxeudo Suite software (Illumina) was used for analysis;

including TopHat (version 2.0.13) and Bowtie2 (version 2.2.1) for alignment to the UCSC hg19 reference genome, FastQC for a second round of quality control, and Cufflinks (version 2.2.1) for differential expression analysis. Only sequences that aligned to the human (rather than mouse) genome were used for analysis. The 117 genes with a q value of < 0.05 for differential expression (involved vs. uninvolved) in either positive or negative direction were used for pathway analysis using DAVID version 6.8 software and the KEGG database. This dataset is publicly available in GEO under accession number GSE186061. All experimental procedures were approved by the University of Michigan Animal Care and Use Committee, protocol #PRO00007427.

Du-145 Xenograft RNAseq Results: To discover new genes and pathways potentially involved in prostate cancer dormancy and recurrence, we compared gene expression from prostate cancer cells isolated macroscopic experimental bone tumors (actively growing) vs. prostate cancer cells isolated from bones of the same animals with no grossly detectable tumors (dormant). Tumors were detected by the presence or absence of bioluminescence signal from luciferase labeled human Du145 cells in SCID mice (Supp. Fig. 4) Samples and FACS isolation methods were from our prior study (Cackowski et al., 2017). We used DAVID version 6.8 software and the KEGG database to perform pathway analysis of genes differentially expressed in either the positive or negative direction and saw significant enrichment in the following pathways; arrhythmogenic right ventricular cardiomyopathy, TGF-beta signaling pathway, amyotrophic lateral sclerosis, basal cell carcinoma, and hippo signaling pathway (Supp. Fig. 4B). We interpreted the enrichment of TGF-beta signaling components as proof of concept for our approach as this pathway is known to be involved in regulation of prostate cancer dormancy (Bragado et al., 2013).

Appendix 3: Additional Detailed Methods:

Western Blotting (Supplemental Fig. 2)

Antibodies used: anti-mouse Rb2/p130 (BD Biosciences, 610261,1:1000), anti-phospho-RB (Cell Signaling, #9308), anti-mouse phospho-Cdk2^{T160} (Cell Signaling, 1:500), anti-mouse Cdk2(Santa Cruz, M2, 1:1000), anti-rabbit p27 (Cell Signaling, #2552, 1:1000) or anti-mouse GAPDH (Cell Signaling, 14C10, 1:2000) were used as loading controls with the appropriate

HRP-conjugated secondary antibody. Enhanced Chemi-luminescence detection (Amersham) was performed and band signal intensity was quantified using NIH Image J.

Flow Cytometry analysis and FACS

For flow cytometry analysis of DNA content, cells were fixed with 4% paraformaldehyde, and then stained with the FxCycle Violet DNA Stain (Thermo Fisher, F10347). Resuspended cells were analyzed on an Attune flow cytometer using the violet laser for DNA measurements. For cell sorting and assays in Supp. Figs. 2 and 4, cells were cultured in DMEM supplemented with either 10% FBS or 1%FBS for 24-72 hr, then subpopulations were sorted according to the intensity of their fluorescent reporters, using a BD FACS Aria II system. Cells were sorted into SDS-PAGE loading buffer or TRIZOL for immediate protein extraction or RNA isolation. A minimum of ~10^5 cells were collected for each experiment.

RT-qPCR

For RT-qPCR, RNA was isolated from sorted cells per the TRIzol manual, resuspended in water, and then treated with TURBO™ DNase to remove contaminating DNA. Using 500ng of RNA per sample, cDNA was synthesized using oligo$(dT)_{20}$ and the Superscript III First-Strand Synthesis System (Invitrogen 18080051). qPCR using 0.5µL of cDNA per reaction was then performed using the 7500 Fast Real-Time PCR and StepOnePlus Real-Time PCR Systems (Applied Biosystems)

Primer sequences

Primer name	Sequence
Forward-GAPDH	ATGTGTCCGTCGTGGATCTGA
Reverse-GAPDH	TTGAAGTCGCAGGAGACAACCT
Forward-PCNA	TTTGAGGCACGCCTGATCC
Reverse-PCNA	GGAGACGTGAGACGAGTCCAT
Forward-Mki67	CCTTGGCTTAGGTTCACTGTCC

Reverse-Mki67	TGCAGAATCCAGATGATGGAGC
Forward-Geminin	GGGAGCCCAAGAGAATGTGAA
Reverse-Geminin	CAAGCCTTTTGGCAACTCATTT
Forward-SOD3	CCTTCTTGTTCTACGGCTTGC
Reverse-SOD3	GCGTGTCGCCTATCTTCTCAA
Forward-PDCD4	CCACTGACCCTGACAATTTAAGC
Reverse-PDCD4	TTTTCCGCAGTCGTCTTTTGG

References for Supplemental Methods

Akaike, H. (1974). A new look at the statistical model identification. *IEEE Transactions on Automatic Control* 19**,** 716-723.

Bragado, P., Estrada, Y., Parikh, F., Krause, S., Capobianco, C., Farina, H.G., Schewe, D.M., and Aguirre-Ghiso, J.A. (2013). TGF-beta2 dictates disseminated tumour cell fate in target organs through TGF-beta-RIII and p38alpha/beta signalling. *Nat Cell Biol* 15**,** 1351-1361.

Cackowski, F.C., Eber, M.R., Rhee, J., Decker, A.M., Yumoto, K., Berry, J.E., Lee, E., Shiozawa, Y., Jung, Y., Aguirre-Ghiso, J.A., and Taichman, R.S. (2017). Mer Tyrosine Kinase Regulates Disseminated Prostate Cancer Cellular Dormancy. *J Cell Biochem* 118**,** 891-902.

Cappell, S.D., Chung, M., Jaimovich, A., Spencer, S.L., and Meyer, T. (2016). Irreversible APC(Cdh1) Inactivation Underlies the Point of No Return for Cell-Cycle Entry. *Cell* 166**,** 167-180.

Cox, D.R. (1972). Regression Models and Life-Tables. *Journal of the Royal Statistical Society. Series B (Methodological)* 34**,** 187-220.

Kaplan, E.L., and Meier, P. (1958). Nonparametric-Estimation from Incomplete Observations. *Journal of the American Statistical Association* 53**,** 457-481.

Naetar, N., Soundarapandian, V., Litovchick, L., Goguen, K.L., Sablina, A.A., Bowman-Colin, C., Sicinski, P., Hahn, W.C., Decaprio, J.A., and Livingston, D.M. (2014). PP2A-Mediated Regulation of Ras Signaling in G2 Is Essential for Stable Quiescence and Normal G1 Length. *Mol Cell* 54**,** 932-945.

Pinheiro, J., Bates, D., Debroy, S., Sarkar, D., and Team}, R.C. (2017). "Linear and Nonlinear Mixed Effects Models", in: *https://CRAN.R-project.org/package=nlme*.).

Sun, D., and Buttitta, L. (2015). Protein phosphatase 2A promotes the transition to G0 during terminal differentiation in Drosophila. *Development* 142**,** 3033-3045.

Tukey, J.W. (1949). Comparing Individual Means in the Analysis of Variance. *Biometrics* 5**,** 99-114.
